# Stanozolol promotes osteogenic gene expression and apposition of bone mineral *in vitro*

**DOI:** 10.1590/1678-7757-2018-0014

**Published:** 2018-11-08

**Authors:** Giulia Ghiacci, Simone Lumetti, Edoardo Manfredi, Daniele Mori, Guido Maria Macaluso, Roberto Sala

**Affiliations:** 1 Università degli Studi di Parma, Dipartimento di Medicina e Chirurgia, Centro Universitario di Odontoiatria, Parma. Italy. Università degli Studi di Parma, Dipartimento di Medicina e Chirurgia, Centro Universitario di Odontoiatria, Parma. Italy; 2 Università degli Studi di Parma, Dipartimento di Medicina e Chirurgia, Unità di Patologia Generale, Parma. Italy; 3 Istituto dei Materiali per l'Elettronica ed il Magnetismo (IMEM) - CNR, Parma. Italy

**Keywords:** Osteogenesis, Bone matrix, Calcification, Gene expression, Androgens, Stanozolol

## Abstract

Stanozolol (ST) is a synthetic androgen with high anabolic potential. Although it is known that androgens play a positive role in bone metabolism, ST action on bone cells has not been sufficiently tested to support its clinical use for bone augmentation procedures. Objective: This study aimed to assess the effects of ST on osteogenic activity and gene expression in SaOS-2 cells. Material and Methods: SaOS-2 deposition of mineralizing matrix in response to increasing doses of ST (0-1000 nM) was evaluated through Alizarin Red S and Calcein Green staining techniques at 6, 12 and 24 days. Gene expression of runt-related transcription factor 2 (RUNX2), vitamin D receptor (VDR), osteopontin (SPP1) and osteonectin (ON) was analyzed by RT-PCR. Results: ST significantly influenced SaOS-2 osteogenic activity: stainings showed the presence of rounded calcified nodules, which increased both in number and in size over time and depending on ST dose. RT-PCR highlighted ST modulation of genes related to osteogenic differentiation. Conclusions: This study provided encouraging results, showing ST promoted the osteogenic commitment of SaOS-2 cells. Further studies are required to validate these data in primary osteoblasts and to investigate ST molecular pathway of action.

## Introduction

The research for new strategies and materials to enhance bone repair and/or bone regeneration is a major goal for the management of demanding clinical cases in orthopedics and maxillofacial surgery.

Androgens (or androgenic hormones) can be defined as any natural or synthetic steroid that stimulates or controls the development and maintenance of primary and secondary male characteristics in vertebrates by binding to the androgen receptor AR. ^1^ Androgens also provide anabolic functions, which result in growth and differentiation of cells and increase in body size. ^2^ Particularly, they play a significant role in regulating skeletal morphogenesis and maintaining bone homeostasis throughout life. ^3^ ^,^ ^4^ The most abundant circulating androgen in men is testosterone, whose effect in peripheral tissues not only depends on a direct action, but also results from a local enzymatic conversion in different metabolites. 5α-reductase and aromatase are among the most important enzymes responsible for testosterone transformation in bone tissues. 5α-reductase activity reflects in the formation of the potent androgen dihydrotestosterone, while aromatase catalyzes androgen conversion into the estrogen estradiol. Depending on its peripheral conversion, systemically administered testosterone may bind either to the AR (testosterone itself or dihydrotestosterone) or to the estrogen receptors ERα/ERβ (testosterone converted to estradiol), which results in androgenic or estrogenic effects. ^5^ ^-^ ^7^

The anabolic potential of androgens leads to the synthesis of molecules with a low androgenic and high anabolic action, with prolonged activity compared with endogenous androgens: these synthetic testosterone-derivative drugs are generally known as anabolicandrogenic steroids (AAS). One of these agents is stanozolol (ST), a non-aromatizable AAS derived from dihydrotestosterone.

Systemic administration of AAS in animal models provided some encouraging results, showing an overall increase in bone formation and mineralization, as well as improvements in bone density and biomechanical properties. ^8^ ^-^ ^10^ Nevertheless, other investigations reported qualitative alterations in the bone geometry and low bone turnover in response to ST treatment. ^11^ In brief, the overall efficacy and the long-term safety of AAS administration for the osteoporosis therapy and the prevention of fracture risk appears to be atleast questionable. ^12^ Systemic administration, local applications of ST and other AAS have been tested in animal models to improve bone healing. Such approaches allow the use of relatively low doses of steroid and imply short-term treatment protocols. Intra-articular ST administration showed positive effects on the synovial membrane and cartilage regeneration in osteoarthritis conditions ^13^ , and ST- soaked deproteinized bone grafts enhanced new bone formation in calvarial critical-size defects. ^14^

Although some evidence has been provided in human and animal studies, only a limited number of studies investigating ST effects on bone cells are currently available. SaOS-2 (literally "Sarcoma OSteogenic") cell line represents a validated option for the study of osteoblastic differentiation and responsiveness to exogenous stimuli. In 1987, Rodan, et al. first conducted a study on SaOS-2 characterization and assessed that these cell lines possess several osteoblastic features and could be useful as a permanent line of human osteoblast-like cells and as a source of bone-related molecules. ^15^

SaOS-2 cells have the advantage of following the main molecular steps of osteoblast differentiation and have the ability "to deposit a mineralization-competent extracellular matrix". ^16^ Thus, they have been recently validated as a feasible model to investigate osteoblast activity and maturation. ^17^ Immunocytochemical assays revealed that SaOS-2 cells express osteoblast-like markers such as osteocalcin (OC or BGLAP) and osteopontin (OPN or SPP1). Expression of genes involved in osteoblast differentiation and function (i.e. runt-related transcription factor 2, RUNX2) has been documented. ^18^ Also, the literature data provided evidence of SaOS-2 responsiveness to steroid stimulation. ^19^

The aim of this study was to assess the effects of ST on osteogenic activity and gene expression in SaOS-2 cells. The investigation of ST effects on bone cells may in fact provide evidence to support the clinical use of this steroid in the field of bone healing and regeneration, particularly for developing targeted drug administration protocols applied to orthopedic, maxillofacial and oral surgery.

## Materials and methods

### Stanozolol preparation

ST powder (ACME Srl, Reggio Emilia, Italy) was weighted and dissolved in absolute ethanol (ETOH), preparing 1000X stock solutions. Sequential dilutions of stocks were performed in the osteogenic medium, to obtain final concentrations of 1 nM, 10 nM, 100 nM, 500 nM and 1000 nM, respectively.

### Cell culture

We preliminarily assessed ST effects on cell proliferation using resazurin assay up to 12 days of culture.

SaOS-2 cells ranging from 8 to 12 passages were plated at a density of 1×10× cells/cm^2^ into 6-well and 24-well plates, using respectively 2 mL and 500 μL of DMEM-low glucose with 10% fetal bovine serum (FBS), penicillin (100 μg/mL), streptomycin (100 μg/ mL) and L-glutamine (2 mM). After 24 h, this medium was replaced with an osteogenic medium consisting of DMEM-low glucose completed with 2-Phospho-L-ascorbic acid (100 μM), L-proline (34.8 μM) and β2-glycerol phosphate (5 mM). The day after (day 0), the medium was changed with fresh osteogenic medium containing stanozolol at the described concentrations, while osteogenic medium with 0.1% ETOH was used as a control. The culture medium was changed every two/three days.

### Culture staining

After 6, 12 or 24 days, cells lying in 24-well plates were treated either with Alizarin Red S or Calcein Green staining.

Alizarin Red S staining: the cells were washed three times with PBS and fixed by adding 250 μL of 4% formaldehyde solution for 15 min at room temperature and rinsed twice with ddH_2_O. Then, 500 μL of Alizarin Red S solution in water (40 mM, pH 4,2) were added to each well, and the whole plates were kept at RT for 30 min with gentle shaking. The dye was removed, and cells were rinsed 5 times (5 min each time) with ddH_2_O.

To measure Alizarin Red S concentration, each well was treated with 200 μL of 10% acetic acid and incubated for 30 min at RT with shaking. Cells were scraped from the plate and transferred to a 1.5 mL microcentrifuge tube and sealed with parafilm. After vortexing vigorously for 30 seconds, the samples were heated to 85°C for 10 min. Then they were transferred on ice for 5 min and centrifuged at 20000 rpm for 15 min. After centrifugation, the slurry was transferred to a new tube, and pH was adjusted to 4.1-4.5 by adding 75 μL of 10% ammonium hydroxide. An Alizarin Red S standard curve was prepared with serial dilutions of Alizarin Red ranging from 10 mM to 10 μM, absorbance was measured at 405 nm with an Enspire microplate reader (Perkin Elmer, Waltham, Massachusetts, USA).

Calcein Green staining: 24 h before the end of the experimental period, 2 μl of Calcein Green (10 mg/mL) were added to each well. At the end of the experimental period, the samples were treated with 500 μl of acetic acid 10% dabbed with ammonium hydroxide pH 7.0. The whole plate was placed under slow oscillation for 20 min and then placed in an ultrasonic bath for 15 min. Each well was then washed three times with PBS. Semi-quantitative analysis of Calcein Green fluorescence was measured with an Enspire microplate fluorescence reader (Perkin Elmer, Waltham, Massachusetts, USA) set to a wavelength of 512 nm, as described elsewhere. ^20^

### Gene expression analysis

RNA extraction and reverse transcription: At 12 and 24 days of culture, total RNA was isolated from cells seeded onto 6 well dishes with GenEluteTM Mammalian Total RNA Miniprep Kit (Sigma-Aldrich) following the manufacturer's instructions, and 1 μg RNA/sample was reverse transcribed to cDNA (GoScript Reverse Transcription System, Promega Corporation, Madison, Wisconsin, USA). Briefly, RNA on 0.5 μg of random hexamer oligonucleotide primers, in a total volume of 5 μΙ, was heated to 70°C for 5 min, cooled to 4°C for 5 min, and then incubated with 15 μl of a mixture of components to achieve the final concentration of 0.5 mM each dNTPs, 1× first-strand buffer, 3 mM MgCl_2_, 1 U/μI Recombinant RNasinR Ribonuclease Inhibitor, Improm-II 1 μl/reaction, for 1 h at 42°C. The reaction was stopped by heating to 70°C for 15 min. The RT reaction was then diluted with nuclease free water to a total volume of 200 μl, and a triplicate of 5 μl aliquots was used for gene expression quantification in a 20 μl PCR.

**Figure 1:**
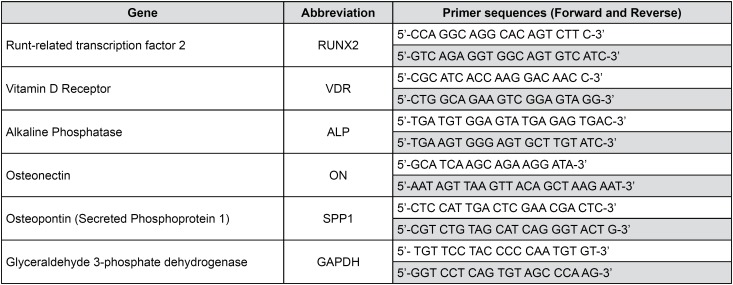
Sequences of primers used for RT-PCR

Polymerase chain reaction: The primer set was designed according to the known sequences reported in GenBank with Primer 3 program [Steve Rozen, Helen J. Skaletsky (1998) Primer3. Code available at http://www-genome.wi.mit.edu/genome_software/other/primer3.html .] ( Figure 1 ). cDNA was amplified with 1X GoTaq qPCR Master, 5 pmol specific primers and RNase-free water. PCR was performed in a 36- well Rotor Gene 3000 (Rotor-Gene™ 3000, version 5.0.60, Mortlake, Australia). Each cycle consisted of a denaturation step at 95°C for 15 s, followed by separate annealing (15 s, 57°C or 60°C, depending on the examined gene) and extension (15 s, 72°C) steps. Fluorescence was monitored at the end of each extension step. A no-template, no-reverse transcriptase control was included in each experiment. At the end of the amplification cycles a melting curve analysis was added. The data analysis was performed according to the Relative Standard Curve Method. ^21^ Data normalization was carried out in relation to the housekeeping gene glyceraldehyde 3-phosphate dehydrogenase (GAPDH), which was found to be expressed uniformly in all the tested conditions.

### Statistical analysis

Growth curves were analyzed using the Boltzmann sigmoidal function, and a comparison of curve fits was performed to verify the null hypothesis of one curve fitting all data sets and the alternative hypothesis of different curves for each culture condition. Cell differentiation and osteogenic activity was analyzed with one-way ANOVA and Tukey's post-test. A linear regression analysis was performed to assess variations on different time-points. p<0.05 was considered the level of statistical significance. Graphs were obtained with GraphPad Prism 6.0 software. Data are expressed as mean value ± standard deviation.

## Results

### Culture staining

**Figure 2:**
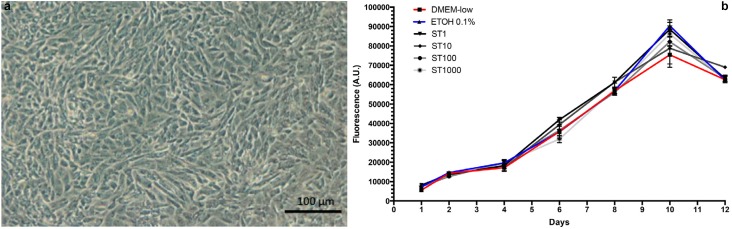
(a) Aspect of SaOS-2 cells at confluency. Optical microscopy, 10X magnification; (b) Graphic representation of SaOS-2 growth under different conditions: DMEM-low (red line), ETOH 0.1% (blue line), ST 1-1000 nM (shades of grey). The X axis represents the days of culture, whereas the Y axis reports fluorescence values expressed in arbitrary units (A.U.)

Optical microscopy showed a typical polygonal shape of SaOS-2, which tended to become slightly elongated once they reached confluence. The resazurin assay revealed a growth pattern perfectly fitting a Sigmoidal Boltzmann curve up to 10 days of culture (DMEM low: r^2^=0.94, ETOH 0.1%: *r^2^* =0.96, ST 1nM: *r^2^=0.98,* ST 10nM: *r^2^* =0.93, ST 100nM: *r^2^* =0.99, ST1000 nM: *r^2^* =0.98), while at 12 days of culture an overall decrease in cell vitality was recorded independently of the culture conditions. A comparison of curve fits did not allow us to reject the null hypothesis of one curve fitting all data sets (p=0.8), thus indicating a superimposable growth pattern of SaOS-2 under all the tested conditions up to the end of the experimental period. A graphic representation of data is reported in Figure 2 .

**Figure 3:**
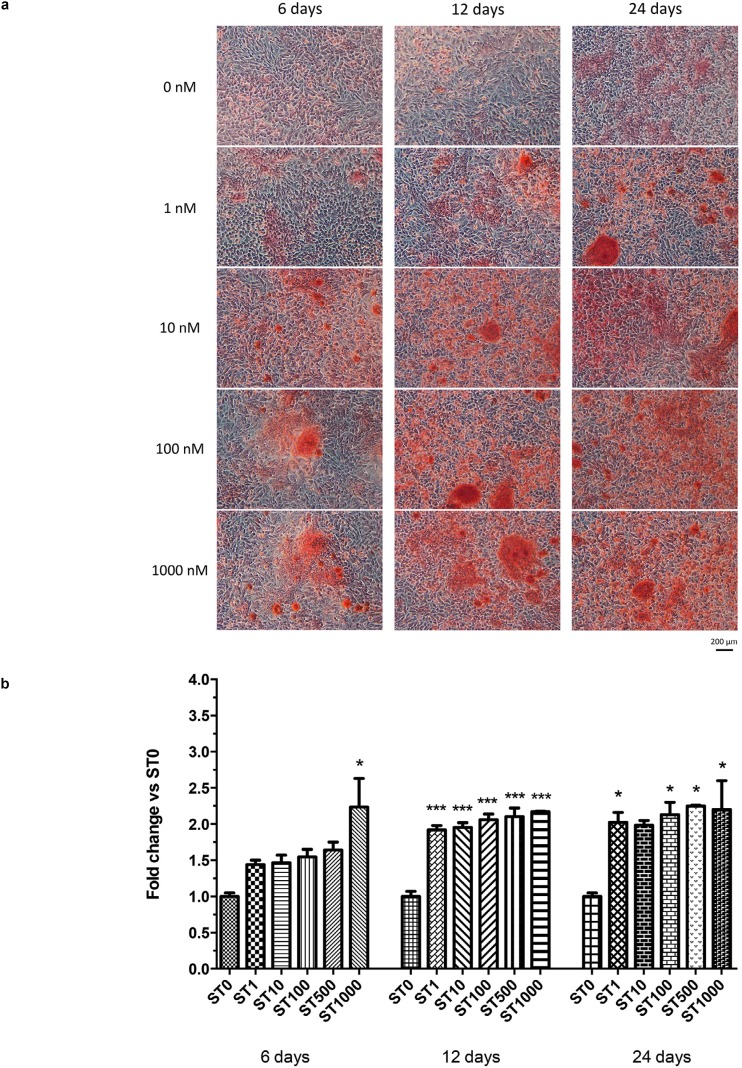
(a) Appearance of SaOS-cell culture treated with different stanozolol (ST) concentrations (0-1000 nM) at 6, 12 and 24 days after Alizarin Red S staining. Optical microscopy, 10X magnification; (b) Alizarin Red S staining quantification with different ST concentrations (0-1000 nM) at 6, 12 days and 24 days. Data are reported as fold change over controls and expressed as mean ± standard deviation. Asterisks indicate statistical significance (*: p<0.05 vs ST 0 nM; ** p<0.005 vs ST 0 nM; ***: p<001 vs ST 0 nM)

Alizarin Red S staining confirmed the capacity of SaOS-2 to produce calcified extracellular matrix. The apposed matrix was characterized by round-shaped granules which increased progressively both in size and in number depending on the concentration of the administered steroid and extent of the induction ( Figure 3 a). Cells treated with ST revealed the presence of areas with mineralization since the earlier observation time-point, which peaked at 1000 nM concentration (fold change vs control: ST 1 nM: 1.44±0.08, p>0.05, ST 10 nM: 1.47±0.15, p>0.05, ST 100 nM: 1.55±0.15, p>0.05, ST 500 nM: 1.64±0.16, p>0.05, ST 1000 nM: 2.24±0.56, p<0.05). At 12 days, SAOS cell layers cultured with ST appeared consistently more filled with calcified granules compared with the controls at all the tested doses (fold change vs control ST 1 nM: 1.92±0.08; ST 10 nM: 1.95±0.09; ST 100 nM: 2.06±0.11; ST 500 nM: 2.10±0.16; ST 1000 nM: 2.17±0.01, p<0.01). A similar outcome was recorded at 24 days (fold change vs control ST 1 nM: 2.02±0.19; ST 100 nM: 2.13±0.24; ST 500 nM: 2.25±0.01, ST 1000 nM: 2.20±0.57, p<0.05) ( Figure 3 b).

**Figure 4:**
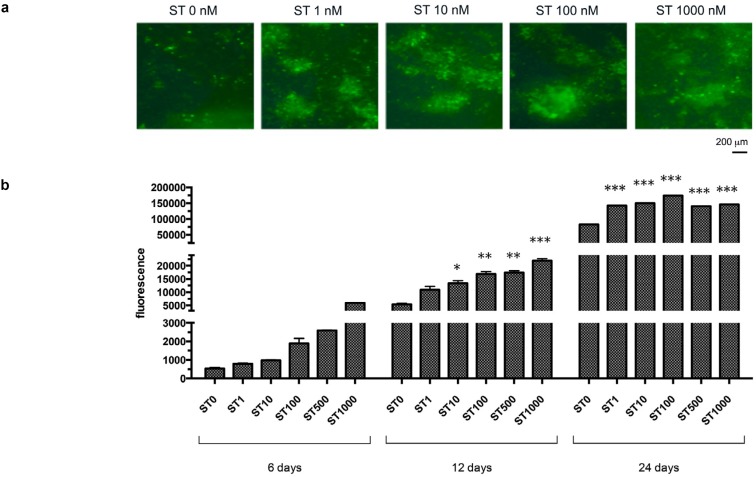
(a) Appearance of samples treated with stanozolol (ST) (0-1000 nM) at 24 days observation period using a phase contrast microscopy and fluorescence microscopy to reveal Calcein Green staining (10X magnification); (b) Graph illustrating fluorescence absorbance of Calcein Green staining with different ST concentrations (0-1000 nM) at 6, 12 and 24 days observation period. Data are expressed as mean ± standard deviation. Asterisks indicate statistical significance (*: p<0.05 vs ST 0 nM; ** p<0.005 vs ST 0 nM; ***: p<001 vs ST 0 nM)

Semiquantitative analysis of Calcein Green fluorescence revealed a deposition of calcium phosphates in response to ST administration ( Figure 4 a). At 6 days’ observation, a dose-dependent trend was also evident (fold change vs control ST 1nM: 1.50±0.16, p>0.05; ST 10 nM: 1.84±0.18, *p* >0.05; ST 100 nM: 3.58±0.54, *p* <0.005; ST 500 nM: 4.89±0.46, *p* <0.01; ST 1000 nM: 11.27±1.06, *p* <0.01). Observations at further time-points revealed a massive calcification in all the samples. All the tested ST doses produced significantly higher Calcein Green fluorescence compared with the controls both at 12 days (fold change vs control ST 1 nM: 2.03±0.14, *p* <0.05; ST 10 nM: 2.46±0.21, *p* <0.05; ST 100 nM: 3.11±0.21, p<0.005; ST 500 nM: 3.21±0.21, p<0.005; ST 1000 nM: 4.04±1.06, p<0.05) and 24 days (fold change vs control ST 1 nM: 1.75±0.10; ST 10 nM: 1.80±0.04; ST 100 nM: 2.07±0.04; ST 500 nM: 1.67±0.04; ST 1000 nM: 1.74±0.04; p<0.01) ( Figure 4 b).

### Gene expression analysis

**Figure 5:**
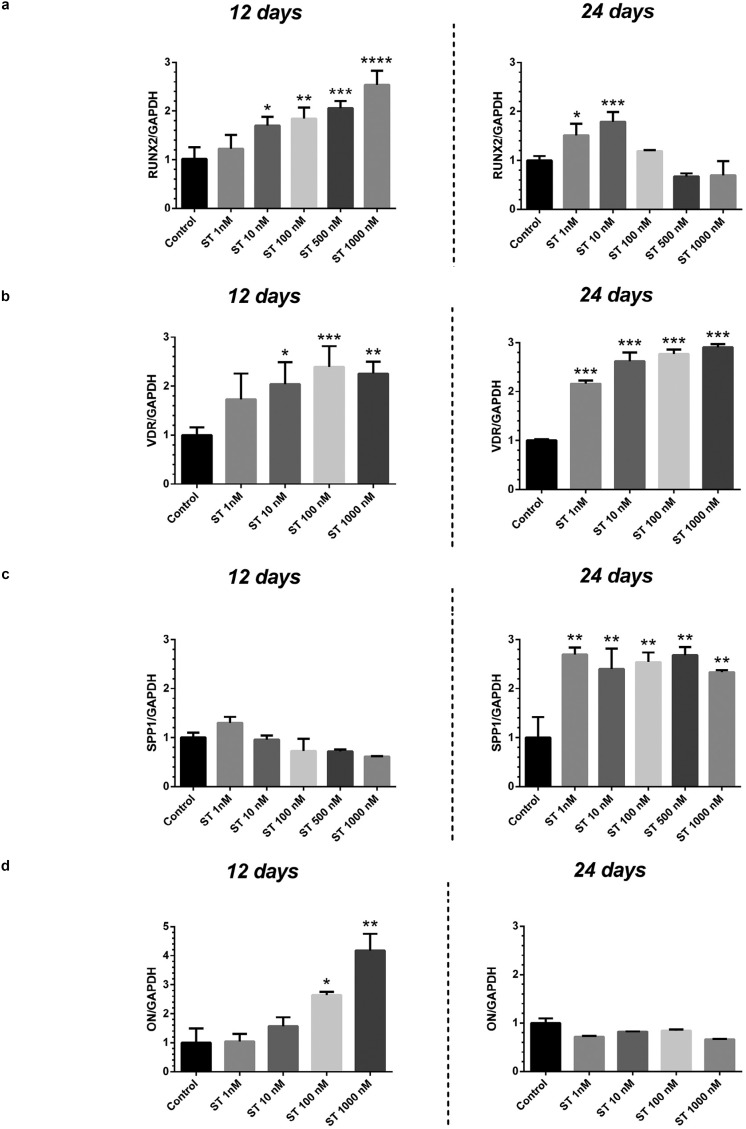
Gene expression of SaOS-2 treated with different concentrations (0-1000 nM) of stanozolol (ST) at 12 and 24 days observation period; (a) RUNX2: runt-related transcription factor 2; (b) VDR: Vitamin D Receptor; (c) SPP1: Osteopontin; (d) ON: Osteonectin. Data are reported as fold change over 0 nM ST and are expressed as mean ± standard deviation. Asterisks indicate statistical significance (*: p<0.05 vs control; ** p<0.005 vs control; ***: p<001 vs control)

The gene expression analysis related to osteogenic differentiation revealed differences depending both on the time-point (either 12 or 24 days) and on the concentration of the steroid ( Figure 5 ).

RUNX2: At 12 days’ observation, the Runx2 expression was shown to increase at growing concentrations of ST, with significant differences vs controls for doses ranging from 10 to 1000 nM (fold change vs control ST 10 nM: 1.701±0.182, *p* <0.05; ST 100 nM: 1.847±0.226, p<0.005; ST 500 nM: 2.061±0.143, *p* <0.001; ST 1000 nM: 2.535±0.295, *p* <0.001). A similar pattern was recorded at 24 days (fold change vs control ST 1 nM: 2.025±0.191; ST 100 nM: 2.130±0.240; ST 500 nM: 2.250±0.014, ST 1000 nM: 2.200±0.566, *p* <0.05). At 24 days, the Runx2 expression showed a significant increase vs control only at the lowest ST concentrations (1 and 10 nM)used (fold change vs control ST 1nM: 1.514±0.234, *p* <0.05; ST 10 nM: 1.786±0.201, *p* <0.005). A tendency to decrease at the highest ST concentrations (500, 1000 nM) was also detected, although without any statistical significance ( Figure 5 a).

VDR: The VDR expression showed a consistent increase vs controls with the administration of the highest ST concentrations (fold change vs control ST 10 nM: 2.037±0.543, *p* <0.05; ST 100 nM: 2.388±0.427, *p* <0.001; ST 1000 nM: 2.255±0.247, *p* <0.001) at 12 days. At 24 days, all the tested ST doses were associated with significantly higher VDR expression vs controls (fold change vs control ST 1 nM: 2.158±0.070; ST 10 nM: 2.622±0.179; ST 100 nM: 2.770±0.090, ST 1000 nM: 2.901±0.073, *p* <0.001) ( Figure 5 b).

SPP1: The expression pattern of SPP1 showed variations depending on the observation period, with no significant differences in test groups vs controls at 12 days ( *p* >0.05) and a consistent induction observed at 24 days for all the tested concentrations of ST (fold change vs control ST 1 nM: 2.691±0.145; ST 10 nM: 2.401±0.416; ST 100 nM: 2.540±0.197; ST 500 nM: 2.680±0.166, ST 1000 nM: 2.331±0.048, *p* <0.005) ( Figure 5 c).

ON: The ON gene expression increased in response to the higher ST dose of 100 nM (fold change vs control: 2.645±0.109, *p* <0.05) and 1000 nM (fold change vs control: 4.175±0.577, *p* <0.001) at 12 days. At 24 days, no significant differences in test groups vs controls were recorded ( *p* >0.05) ( Figure 5 d).

## Discussion

This research investigated the effects of different doses of ST on the proliferation and osteogenic response of SaOS-2 cells. Growing evidence suggests androgens act directly on bone cells, playing a complex regulatory role. ^22^ Androgen effects on osteogenic differentiation are still controversial, nevertheless it has been suggested they may stimulate osteoblastic differentiation and extracellular bone matrix apposition. ^23^ ^-^ ^25^ Previous authors observed the effects of androgenic steroids on cell lines and reported positive effects of testosterone at doses of 10^-10^ M and 10^-9^ M on the proliferation of SaOS-2 cells after 48 h. ^26^ However, to the best of our knowledge, only one study reported on ST effects on osteogenic activity of bone cells, concluding that "Stanozolol at a concentration of 10^-10^ mol/l to 10^-6^ mol/l consistently stimulated the incorporation of [ ^3^ H]thymidine into DNA of human bone cells and increased proliferation" up to 15 days of culture. ^27^

According to our assay, ST treatment at the doses of 1 to 1000 nM did not affect the growth pattern of SaOS-2 cells up to 12 days of culture. This result may be due to the specific characteristics of the steroid used, although a peculiarity of the cells used in our experimental setting cannot be ruled out. Indeed, various SaOS-2 subpopulations that responded differently to proliferative and differentiative stimuli were identified. ^28^ Moreover, the phenotypic stability of SaOS-2 may be affected by the number of passages they have undergone: it was noticed that a higher passage SaOS-2 demonstrated higher proliferation rates and lower alkaline phosphatase activities, although mineralization was significantly more pronounced in cultures of late passage cells. ^29^ Such findings are consistent with our results of an overall high proliferation rate of SaOS-2 ranging from 8 to 12 passages as well as a high mineralizing activity.

Alizarin Red S and Calcein Green staining showed ST administration notably increased mineralization. These findings highlighted the advantages of treating cells with androgens compared with the use of a standard differentiation medium. At 12 days’ observation all the tested doses showed a similar effect with Alizarin Red S quantification technique, whereas a different dose- dependent effect was recorded with Calcein Green staining. These differences may point to a greater sensitivity of Calcein Green technique compared with Alizarin Red S. Nevertheless, neither Alizarin Red S nor Calcein Green revealed any differences between the effect of treatment at 24 days’ observation, when all the samples presented abundant uniform calcification.

RT-PCR analysis revealed a modulatory role played by ST on the gene expression related to osteogenic differentiation. RUNX2 represents an early differentiation marker, as its expression is enhanced since the first stages of osteoblast maturation. ^30^ The detection of RUNX2 mRNA in control samples confirmed previous observations that described a constitutive expression of this gene in SaOS-2 cells. ^18^ In addition, we found out that RUNX2 expression may be modulated by steroid treatment: according to our results at 12 days, the expression of RUNX2 was increasing with a dose-dependent trend, consistently with the mineralization pattern (Calcein Green staining). We may hypothesize that treatment with higher doses of ST induced a faster activation in terms of osteo-differentiation and mineralization when compared with lower doses. Thus, an overall decrease in RUNX2 expression at 24 days in samples treated with high doses of ST is compatible with a lower mitotic activity and a more mature phenotype. On the other hand, lower doses may produce a similar effect throughout a longer timeframe. It would be interesting to investigate the mineralization pattern occurring between 12 and 24 days, as at 24 days we observed a massive mineralization, which may mask previous differences between samples.

Another hypothesis to explain RUNX2 decrease at 24 days is that of a biphasic effect of higher ST doses, which may improve cell differentiation at early time points (12 days) and may not keep this effect at late time points (24 days). A biphasic effect of androgens on cell viability has been described in the literature, with an initial increase in cell proliferation followed by a decrease after prolonged exposure. ^31^ However, according to our preliminary assay, ST treatment did not affect the growth pattern of SaOS-2 up to 12 days. It would be interesting to investigate whether a different effect on cell viability is observed between 12 and 24 days.

An increase in SPP1 expression in response to ST was recorded respectively at 12 and 24 days of ST treatment, which first demonstrated the modulatory activity of this androgen on genes related to osteogenic function. Interestingly, the expression pattern of RUNX2 and SPP1 was shown to be inversely correlated, with a marked increase of SPP1 observed together with a decrease in RUNX2 expression. This finding may indicate an expression switch from 12 to 24 days, as it was observed that in SaOS-2 cells RUNX2 repressed SPP1 gene expression, and the induction of SPP1 expression during normal human osteoblast differentiation has been previously related to a decrease in RUNX2. ^32^ Consistently, the ON expression pattern revealed that, at the highest tested concentration, ST promoted the initial phases of osteoblastic commitment (12 days), whereas its action was no more evident at a longer time-point (24 days), when the differentiation was more advanced. Another gene expression that was strongly enhanced by ST treatment in our study was VDR, which encodes the nuclear hormone receptor for vitamin D3 and has been recognized as a key gene for SaOS-2 differentiation elsewhere. ^33^ It would be relevant to assess changes in the expression of other genes typical of both early and late differentiation phases and to set a more complete differentiation profile of cells in response to growing steroid doses. Moreover, an examination of protein levels would be appropriate to validate our RT- qPCR data, since mRNA expression could not directly correlate to protein translation and activity.

A major limitation of this study is represented by the lack of assessment of ST receptor binding and molecular pathway of action. Since ST is a non- aromatizable androgen, we may suppose its action to be exerted through AR. The expression of AR in SaOS-2 cells has been previously described in the literature. ^34^ However, the interaction of ST with AR and its influence on cell transcriptional activity is still unclear: previous studies documented an activation of AR in response to ST treatment, ^35^ but also a variety of other receptors have been reported as ST ligands (including progesterone receptor, estrogen receptor alpha and low-affinity glucocorticoid-binding sites). ^36^ ^-^ ^39^ Such differences could be dependent on the cell type, as ST may have tissue-specific binding sites and elicit differential biological responses. According to these considerations, it would be relevant to characterize SaOS-2 receptor profile, to investigate ST binding to AR and to perform blockage tests to verify the activation of different molecular pathways in response to ST administration.

Finally, we recommend considering potential side effects of AAS in further *in vivo* studies: changes in cholesterol levels (increased low-density lipoprotein and decreased high-density lipoprotein), liver damage, nephropathy, cardiovascular pathologies as well as conditions pertaining to hormonal imbalance have been reported in response to AAS high-dose or prolonged administration. ^40^

## Conclusions

This study provided encouraging results, as it showed ST promoted the osteogenic commitment of SaOS-2 cells, by enhancing the mineralization process and modulating the expression of genes related to osteogenic differentiation.

Nevertheless, further studies are required to validate these data in primary osteoblasts as well as to investigate ST receptor binding and molecular pathway of action.
